# A Systematic Literature Review on the Use of Dried Biofluid Microsampling in Patients With Kidney Disease

**DOI:** 10.1002/jcla.25032

**Published:** 2024-03-25

**Authors:** Megan K. Lamond, Andrew J. Chetwynd, Alan D. Salama, Louise Oni

**Affiliations:** ^1^ Department of Women's and Children's Health, Institute of Life Course and Medical Sciences University of Liverpool Liverpool UK; ^2^ Department of Biochemistry and Systems Biology, Centre for Proteome Research, Institute of Systems, Molecular and Integrative Biology University of Liverpool Liverpool UK; ^3^ Department of Renal Medicine University College London London UK; ^4^ Department of Paediatric Nephrology Alder Hey Children's NHS Foundation Trust Hospital Liverpool UK

**Keywords:** dried blood spot (DBS), dried saliva spot, dried urine spot (DUS), microsampling, renal

## Abstract

**Background:**

Kidney disease is fairly unique due to the lack of symptoms associated with disease activity, and it is therefore dependent on biological monitoring. Dried biofluids, particularly dried capillary blood spots, are an accessible, easy‐to‐use technology that have seen increased utility in basic science research over the past decade. However, their use is yet to reach the kidney patient population clinically or in large‐scale discovery science initiatives. The aim of this study was to systematically evaluate the existing literature surrounding the use of dried biofluids in kidney research.

**Methods:**

A systematic literature review was conducted using three search engines and a predefined search term strategy. Results were summarised according to the collection method, type of biofluid, application to kidney disease, cost, sample stability and patient acceptability.

**Results:**

In total, 404 studies were identified and 67 were eligible. In total, 34,739 patients were recruited to these studies with a skew towards male participants (> 73%). The majority of samples were blood, which was used either for monitoring anti‐rejection immunosuppressive drug concentrations or for kidney function. Dried biofluids offered significant cost savings to the patient and healthcare service. The majority of patients preferred home microsampling when compared to conventional monitoring.

**Conclusion:**

There is an unmet need in bringing dried microsampling technology to advance kidney disease despite its advantages. This technology provides an opportunity to upscale patient recruitment and longitudinal sampling, enhance vein preservation and overcome participation bias in research.

## Graphical abstract

We carried out a systematic review of the use of dried biofluids in kidney disease. The majority of studies focused on drug quantification following transplant. However, dried biofluids were comparable with venous samples opening up the possibility to expand to biomarker discovery.

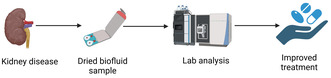

## 1 Introduction

Kidney disease is fairly unique due to the lack of symptoms associated with disease activity, and it is therefore dependent on biological monitoring [1]. Biosampling is an essential and routine part of kidney care that spans patients of all ages from the time of diagnosis to kidney failure, transplantation and beyond. In addition, vein preservation is an important consideration for patients with kidney disease as frequent invasive blood test sampling can lessen the chances of creating a successful fistula for life‐saving dialysis [2, 3]. The adoption of novel sampling techniques for clinical purposes or for research may support vein preservation in this population and provide a more inclusive way to overcome participation bias through accessible home testing [4, 5].

There have been many reported preclinical studies evaluating the methodology for collecting dried biofluid samples [6], mimicking the success of clinical dried blood spots (DBS) such as the heel prick test in newborn screening programs around the world [7, 8]. However, there has yet to be translation from basic science to the incorporation of dried biofluid sampling for clinical benefit or for research advances despite both adult and paediatric populations showing acceptability of this methodology [9]. In 2020, the COVID‐19 pandemic demonstrated that the general public could successfully conduct at‐home sampling, and therefore, exploring this methodology for other conditions is opportune [10, 11]. The technique has many advantages, including enabling sampling at home with postal return providing patient empowerment over when to conduct the test and overcoming barriers to inclusion for patients living more remotely and to those from low socioeconomic backgrounds who may struggle with the costs associated with attending clinical settings [12, 13]. It allows patients to decide when to provide additional samples correlating to clinically significant events such as disease flares or intercurrent infections [11]. There may be many reasons that explain the poor translation into clinical practice such as the requirement to validate and standardise the use of dried biofluids for specific tests or purposes, the stability of the sample, concern over the sensitivity and specificity of the approach when compared to traditional sampling and a lack of evidence that these samples are suitable for large‐scale studies. The aim of this systematic literature review was to describe the currently reported use of dried biofluids in the field of kidney disease.

## 2 Methods

### 2.1 Study Design

This study is a systematic literature review on dried biofluids in kidney disease to produce a summary of the key findings.

### 2.2 Search Strategy

The collation of the relevant literature took place on 12 August 2022 using three databases (PubMed, Web of Science and Scopus). The search strategy used the following terms: (“Dried blood Spot*” OR “Dried urine spot*” OR “Dried sample” OR “Volumetric absorptive microsampling” OR “VAMS” OR “Dried Biofluid” OR “Dried Saliva”) AND (“Patient*” OR “Paed*” OR “Pediatric” OR “Child*” OR “Adult*” OR “adolescen*”) AND (“Renal” Or “kidney” OR “Neph*”). All paper details for each database search were saved on 12 August 2022 prior to the systematic review of this literature.

### 2.3 Eligibility Criteria

Papers were screened according to predetermined eligibility criteria. Studies, which passed the following inclusion criteria, were screened for: (1) presenting primary data focused on humans with kidney diseases; (2) use of dried blood, urine or saliva samples including studies on drugs known to be required for kidney disease or adversely affect the kidneys; (3) available in English language and (4) studies published between 12 August 2012 and 12 August 2022. Exclusion criteria were as follows: (1) ineligible population, (2) wrong study design, (3) not available in full text, (4) not related to kidney research and/or dried biofluid, (5) incorrect outcome and (6) incorrect publication type.

### 2.4 Data Extraction and Reporting

The papers were independently screened by title and then abstract to determine eligibility by two authors (M.K.L. and A.J.C.). After searching the chosen databases, all duplicate studies were eliminated. From each of the included studies, the following data were extracted: the collection method, type of biofluid, application to kidney disease, cost, sample stability and patient acceptability. The results obtained from this systematic review were reported in accordance with the established Preferred Reporting Items for Systematic Reviews and Meta‐Analyses (PRISMA) guidelines.

### 2.5 Statistics

Statistics were reported from the studies identified, and statistical significance was defined as a corrected *p*‐value of 0.05 or lower. Where a correlation analysis was carried out comparing dried biofluids to the conventional liquid form, *R* ^2^ < 0.30 was considered not correlated, 0.30–0.50 a low correlation, 0.50–0.70 moderate correlation, 0.70–0.90 high correlation and 0.90–1.00 very highly correlated [14].

### 2.6 Ethics and Regulatory Approvals

Ethics and regulatory approvals were not required as the study is a secondary review of the existing literature. This study did not qualify for PROSPERO registration because it was not focused on direct patient benefit.

## 3 Results

### 3.1 Search Results

**FIGURE 1:**
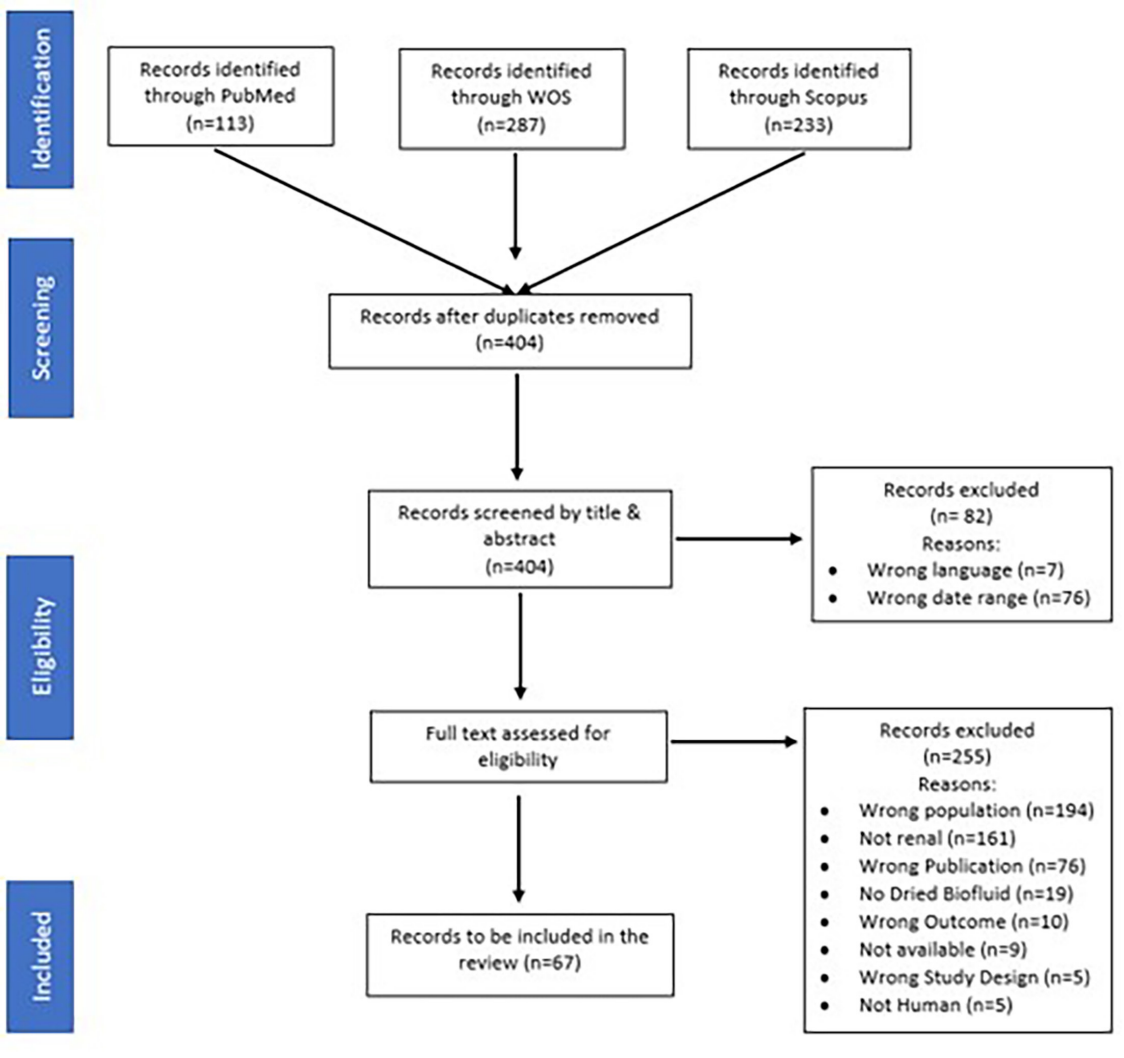
PRISMA diagram showing the inclusion and exclusion of studies of dried biofluids in kidney research.

In total, 633 papers were identified across the three databases (Scopus yielded 233 publications, PubMed 113 and Web of Science 287), and 229 papers were duplicates and consequently removed. The remaining 404 studies were screened against the aforementioned criteria. Six papers were not available in English, and 76 papers were outside the included date range. A total of 322 papers underwent abstract review, resulting in 255 papers excluded and 67 papers for inclusion within the final analysis (Figure 1).

### 3.2 Participants

The 67 included studies involved a total of 34,739 patients. Of these, 31,014 (89%) had gender data reported consisting of 22,654 (73%) male and 8356 (27%) female patients and four transwomen (< 0.1%). Eight studies (12%) did not include demographic data on gender; in addition, one study did not detail the total number of patients. In total, 12 (32%) studies explicitly recruited paediatric patients, totalling 3030 (9%) patients.

### 3.3 Key Extracted Themes

There were six key extracted themes that included the collection method, the type of biofluid, the application to kidney disease, cost, sample stability and patient acceptability.

#### 3.3.1 Collection Method

The majority of the studies described the type of microsampling method used (*n* = 48, 89.6%). The leading method involved a non‐volumetric collection onto filter paper (*n* = 54, 80.5%). Notably, different types of sample filter paper were used: Whatman 903 cards (*n* = 14), Whatman FTA DBS cards (*n* = 7), Whatman FTA DMPK‐A (*n* = 2), Whatman FTA DMPK‐C (*n* = 3) and Guthrie's paper (*n* = 5). Volumetric absorptive microsampling (VAMS) devices were utilised in seven (10%) studies. Three studies (4.5%) compared the VAMS collection method and the nonvolumetric filter paper to establishing the optimal collection method. The results were mixed depending upon the analyte being studied, highlighting a need for improved sample recovery optimisation. Interestingly, 32.3% of VAMS samples were of poor quality due to under‐ or oversaturation of the device or contamination with container compared with 6.2% collected on filter paper [15].

#### 3.3.2 Type of Biofluid Analysed and Comparators

Throughout the 67 studies, three biofluids were analysed: blood from 31,438 participants (*n* = 65, 97%), urine from 3020 participants (*n* = 2, 3%) and saliva from 20 participants (*n* = 1, 1.5%). In one study, the number of participants was not recorded. A number of these studies compared dried biofluid against venous blood. During comparisons for creatinine quantification, 95% of results were distributed within the mean ± 1.96 standard deviations [16], demonstrating a strong correlation in several studies [17, 18, 19].

There was a strong correlation seen when evaluating the glomerular filtration rate (GFR), with 80% of DBS samples being within 10% of the venous measurements [20]; a further study also showed a strong correlation with an *R* of 0.95 (*p* < 0.001) [21]. One study reported that when GFR exceeded 60 mL/min/1.73 m^2^, the DBS overestimated the venous GFR [22]; in contrast, another study found that when GFR was < 60 mL/min/1.73 m^2^, there was diminished accuracy [23]. The impact of non‐volumetric and volumetric DBS samples was assessed [24]. The latter eliminated the effect of varying haematocrit and the need to normalise for sample volume. The study found that only volumetric DBS samples yielded results that correlated with venous plasma samples [24]. Interestingly, one study compared DBS and hair samples and indicated that 87% of hair samples and 84% of DBS samples demonstrated treatment adherence [25]. In the case of dried urine spots, one study compared it to matched urine samples with a moderate correlation (*R* = 0.68 and 0.69, *p* < 0.001) for β2‐microglobulin and β2 microglobulin/creatinine concentration ratios, respectively [26].

#### 3.3.3 Application of Dried Biofluids in Kidney Disease

Dried biofluids were used for a broad range of applications in evaluating kidney diseases including drug monitoring (49%), kidney function assessment (12%), disease activity monitoring (34%) and biomarker discovery (4%).

##### 3.3.3.1 Drug Monitoring

The literature revealed that dried biofluids were predominantly used for monitoring immunosuppressant drug concentrations (*n* = 33, 49.3%) (Table 1, all prospective cohort studies). The majority were monitoring tacrolimus concentration, where paired DBS and venous samples fell within ±20% of each other for 85%–90% of patients regardless of the form of DBS [15, 18, 27, 28, 29, 30, 31, 32, 33, 34, 35, 36, 37, 38, 39, 40, 41, 42, 43, 44, 45]. Only one study found that tacrolimus concentrations using DBS were 22.5% higher than using venous samples [18]. Additional immunosuppressant drugs that were evaluated included cyclosporin A (*n* = 1) with a very strong correlation found between venous and DBS samples (*R* = 0.93, *p* < 0.0001) [18], sirolimus (*n* = 2) where similar results we found between venous and DBS samples in one study [46] while another found a small significant lower concentration in DBS [32], and mycophenolic acid (MPA) (*n* = 4). In the case of MPA, 77% of DBS values fell within ±20% compared with venous blood [33, 47] with a correlation coefficient of > 0.95 [27, 48].

**TABLE 1:** Studies quantifying immunosuppressants from dried blood spots.

Reference	Main aim	Population (age range/mean/median [years]) (sex)	Collection method(s)	Conclusion
[15]	Compare VAMS and DBS samples to whole‐blood venous for tacrolimus concentrations	72 kidney transplant patients (21–78) (42 males [58.3%] and 30 females [41.7%])	VAMS and filter paper	VAMS, compared with filter paper, is inferior for DBS sampling, although both can replace venous sampling
[36]	Compare microsampling techniques to venous blood for tacrolimus, MPA, sirolimus, everolimus, cyclosporine, creatinine and iohexol quantification	25 kidney transplant patients (21–81) (18 males [72%] and 7 females [28%])	VAMS and filter paper	Successfully quantified tacrolimus, MPA and creatinine in DBS and VAMS samples
[38]	Compare microsampling techniques with traditional venous sampling for tacrolimus and creatinine quantification	152 patients	VAMS and filter paper	VAMS is the superior technique for estimating tacrolimus and creatinine
[28]	Compare concentrations of tacrolimus obtained from VAMS with venous samples	82 kidney or liver transplant patients (20–80) (54 males [65.9%] and 28 females [34.15%])	VAMS	VAMS is an alternative technique to venepuncture for tacrolimus quantification
[29]	Evaluate longitudinal tacrolimus concentration	27 patients (mean age = 52) (18 males [67%] and 9 females [33%])	Capillary microsampling	Capillary microsampling suitable for longitudinal tacrolimus quantification
[30]	Quantification of tacrolimus and creatinine in DBS and intravenous samples. Assess patient satisfaction	28 paediatric kidney transplant patients (mean = 13.6 ± 5.4) (18 males [66.7%] and 12 females [42.9%])	Filter paper	DBS can be used to quantify tacrolimus and creatinine
[31]	Determine the feasibility of tacrolimus and creatinine quantification	21 paediatric kidney transplant patients (6–20) (12 males [57.1%] and 9 females [42.9%])	Filter paper	Observed a strong correlation between DBS and the venous samples
[18]	Quantification of tacrolimus, cyclosporin A and creatinine	172 adult kidney transplant patients (20–84) (105 males [61%] and 67 females [39%])	Filter paper	DBS sampling can replace venous sampling for the simultaneous analysis of creatinine and tacrolimus
[32]	Compare venous with DBS samples to quantify sirolimus and tacrolimus	34 paediatric kidney/heart/liver transplant patients (21 males [62%] and 13 females [38%])	Filter paper	DBS can be used interchangeably for the quantification of sirolimus and tacrolimus
[33]	Quantify tacrolimus and mycophenolic acid using DBS samples compared with venous sampling	65 kidney and/or pancreas transplant patients (23–76) (44 males [67.7%] and 21 females [32.3%])	Filter paper	DBS and whole‐blood venous samples are comparable
[34]	Quantify tacrolimus in a paediatric population	39 paediatric kidney or liver transplant patients (4–18) (22 males [56%] and 17 females [44%])	VAMS	VAMS accurately quantified tacrolimus
[27]	Quantify tacrolimus and MPA	28 paediatric patients (2–18) (15 males [54%] and 13 females [46%])	Filter paper	DBS samples can reliably quantify tacrolimus and MPA
[48]	Comparing paired plasma and DBS samples to determine whether they can quantify MPA	19 adult kidney transplant patients	Filter paper	A DBS method can be used to quantify MPA
[47]	Use DBS samples to assess adherence to mycophenolate	33 paediatric kidney transplant patients (8–18) (21 males [63.6%] and 12 females [36.4%])	Filter paper	DBS sampling was deemed ‘convenient, direct approach’ to monitor mycophenolate's adherence
[46]	Validate whether DBS can be used to quantify sirolimus and everolimus in routine care	60 adult transplant patients (23–77) (38 male and [67.9%], 18 females [32.1%])	Filter paper	DBS sampling cannot replace the current venous sampling to quantify sirolimus and everolimus
[19]	Quantify creatinine, tacrolimus, sirolimus, everolimus and cyclosporin A from the same DBS	50 participant samples	Filter paper	DBS is a potential alternative sample to plasma
[35]	Identify the effects of using freeze‐dried blood for the quantification of tacrolimus within DBS samples	10 patients receiving Tacrolimus	Filter paper	The use of freeze‐dried samples on filter paper affects the distribution/spread of blood and thus often results in erroneous results
[37]	Determine the intrapatient variability of tacrolimus concentration	50 adult patients with either kidney or liver transplant (median = 54) (26 males [52%] and 24 females [48%])	Filter paper	Haematocrit levels not a source of the significant bias. DBS and venous sampling returned similar levels
[39]	Compare venous and VAMS samples for tacrolimus quantification	27 kidney transplant patients (21–73) (19 males [70.4%] and 8 females [29.6%])	VAMS	VAMS can quantify tacrolimus concentration reliably after shipping to laboratory
[40]	Quantify tacrolimus from DBS in patients with diarrhoea	24 patients (mean = 53) (11 males [45.8%] and 13 females [54.2%])	Filter paper	DBS samples were successfully used to quantify tacrolimus
[41]	Quantify tacrolimus in DBS	26 kidney transplant patients (mean = 46.5 ± 13.4) (18 males [69.2%] and 8 females [30.7%])	Filter paper	DBS samples were successfully used to quantify tacrolimus
[42]	Determine intrapatient variability in tacrolimus concentrations	40 kidney transplant patients (36–78) (25 males [62.5%] and 15 female [37.5%])	Filter paper	DBS samples were successfully used to monitor tacrolimus levels
[43]	Assess the impact that at‐home DBS sampling has on a patient's travel burden and its effect on societal costs for the quantification of tacrolimus	25 adult kidney transplant patients used for DBS analysis. (52.8 ± 14.1) (17 males [68.0%] and 8 females [32.0%])	Filter paper	At‐home DBS sampling leads to a reduction in a patient's travel burden and the overall costs
[44]	Quantify tacrolimus over a 24‐h period using DBS	26 kidney transplant patients (median = 43.9) (18 males [69.2%] and 8 females [30.8%])	Filter paper	DBS suitable for tacrolimus quantification over a 24‐h period
[45]	Compare the costs associated with tacrolimus, MPA and cyclosporin quantification with DBS and venous sampling in kidney transplant and haemato‐oncology patients	Two patient cohorts, numbers undefined	Filter paper	DBS' at‐home sampling is associated with significant cost saving
[49]	Determine whether DBS and a whole‐blood sample perform equally for everolimus' quantification	20 cancer patients (38–73) (8 males [40.0%] and 12 females [60.0%])	Filter paper	DBS is an accurate/validated method to quantify everolimus

Abbreviations: DBS, dried blood spot; VAMS, volumetric adsorption microsampling.

The analysis of two protein kinase inhibitors (pazopanib [*n* = 1] and everolimus [*n* = 1]), two antidiabetic medications (metformin [*n* = 1] and sitagliptin [*n* = 1]) and the antibiotic ceftriaxone (*n* = 1) was also reported [16, 49, 50, 51]. Only pazopanib showed significant differences between collection methods with a 48% lower concentration reported with matched DBS [50]. Furthermore, to ensure drug adherence and/or safety, three studies quantified tenofovir (a nucleotide reverse transcriptase inhibitor) in DBS [25, 52, 53]. A further study measured both tenofovir and emtricitabine concentrations using a single DBS [53]. Several studies (*n* = 8) utilised DBS for simultaneous creatinine measurement and drug quantification [16, 18, 19, 30, 31, 36, 38]. In this context, Mathew et al. [38] compared between VAMS and filter paper and identified that recovery and reproducibility of creatinine for both devices were excellent with VAMS outperforming filter paper. Finally, one cross‐sectional study established the prevalence of using DBS for monitoring [2‐(2‐nitro‐4‐trifluoromethylbenzoyl)‐1, 3‐cyclohexanedione; nitisinone] (NTBC), a treatment for tyrosinemia type 1, across 21 medical centres. It was noted that of the 21 participating clinics, 38% used DBS to quantify NTBC levels and 62% used DBS to quantify succinylacetone. Furthermore, 10 of the 21 centres determined a patient‐derived full amino acid profile using the DBS [54].

##### 3.3.3.2 Kidney Function Assessment

Dried biofluids were used to formally measure kidney function in eight prospective cohort studies (11.9%) (Table 2). All employed DBS to calculate the GFR at defined time points using contrast agents, iohexol (*n* = 7) or iothalamate (*n* = 1). All studies demonstrated positive findings in support of DBS sampling [20, 21, 22, 23, 24, 55, 56, 57]. It was however reported in one study that patients with a low haematocrit had errors in GFR measurement when using DBS; however, the authors overcame this with the use of a dried plasma sample [21].

**TABLE 2:** Studies utilising dried biofluids to monitor kidney disease.

Reference	Main aim	Population (age range/median or mean [years]) (sex)	Collection method(s)	Conclusion
[17]	Determine whether DBS analysis is suitable for mass screening kidney function	290 paediatric and newborn patients (152 males [52.4%] and 130 females [44.8%])	Filter paper	DBS creatinine values can be used to screen to detect CAKUT
[58]	Evaluate the variability of creatinine concentration between whole‐blood and DBS samples	66 participants (24–88) (20 males [30.3%], 42 females [63.6%] and 4 unrecorded)	Filter paper	Successfully used to determine creatinine concentration
[59]	Estimate the prevalence of Fabry disease	9604 dialysis patients (18–100) (9604 males [100%])	Filter paper	Able to use DBS to screen for Fabry disease by measuring α‐Gal‐A activity and β‐Gal
[60]	Determine the prevalence of Fabry disease in patients presenting with left ventricular hypertrophy	362 patients (19–93) (362 males [100%])	Filter paper	Used DBS to screen for α‐Gal‐A activity and β‐Gal
[61]	Determine the prevalence of Fabry disease in patients who are undergoing dialysis and/or had a kidney transplant	819 end‐stage kidney disease patients (819 males [100%])	Filter paper	An enzymatic assay on DBS samples was able to be used to determine α‐Gal‐A levels
[62]	Assess the prevalence of Fabry disease in a high‐risk population	1012 patients with potential CKD (20–85) (1012 males [100%])	Filter paper	DBS used to assess the activity of α‐Gal‐A alongside neutral α‐glucosidase and β‐Gal
[63]	Identify the prevalence of Fabry disease in CKD patients	1453 CKD patients (mean = 59.3 ± 15.9) (790 males [54.4%] and 663 females [45.6%])	Filter paper	α‐Gal‐A activity and a mutation analysis of the GLA gene could be performed on DBS samples
[64]	Assess the prevalence of Fabry disease within a CKD population who are not undergoing kidney replacement therapy	313 patients with stages 1–5 CKD. (43 ± 14) (167 males [53.4%] and 146 females [46.6%])	Filter paper	DBS can be used to assess α‐Gal‐A activity and determine plasma lyso‐Gl‐3 levels
[65]	Compare the activity of α‐Gal‐A in patients with end‐stage kidney disease and focal segmental glomerulosclerosis	232 patients (FSGS = 43.8 ± 10.4 and ESRD = 61.7 ± 13.9) (122 males [52.6%] and 112 females [48.3%])	Filter paper	DBS samples can be used to assess α‐Gal‐A activity alongside lyso‐Gl‐3 levels. Additionally, they can be used to screen for GLA mutations
[66]	Prevalence of Fabry disease in patients receiving dialysis treatment	526 dialysis patients (325 males [61.8%] and 201 females [38.2%])	Filter paper	α‐Gal‐A activity and a Lyso‐ GB3 assay can be determined/performed on DBS samples
[67]	Evaluate Fabry disease's prevalence in patients with ESRD receiving chronic haemodialysis	5572 patients (3551 males [63.7%] and 2021 females [36.3%])	Filter paper	DBS used to measure α‐Gal‐A activity, as well as assessing globotriaosylsphingosine levels and sequencing the GLA gene
[68]	Assess the prevalence of Fabry disease within patients with CKD using dried urine spots	397 patients (32–75) (279 males [70.3%] and 118 females [29.7%])	DUS and filter paper	DUS can be used to analyse Gb_3_ and creatinine. A GLA gene mutation analysis and α‐Gal‐A activity can also be assessed in DBS samples
[69]	Perform a screening study to determine the presence of Fabry disease	2325 patients (median = 66) (1522 males [65.5%] and 803 females [34.5%])	Filter paper	α‐Gal A can be assessed using DBS samples to diagnose Fabry disease
[70]	Determine the presence of Fabry disease in individuals with CKD (Stages 1–5)	2992 patients (mean = 64) (1732 males [57.9%] and 1260 females [42.1%])	Filter paper	DBS samples can be used to determine α‐Gal A and beta‐galactosidase to diagnose Fabry disease
[71]	Identify the prevalence of Fabry disease within individuals with a reduced GFR	1084 patients with insufficient kidney function (505 males [46.6%] and 579 females [53.4%])	Filter paper	DBS samples can be analysed for α‐Gal A, lyso‐Gl‐3, acarbose‐inhibited acid α‐1,4‐glucosidase and total acid α‐1,4‐glucosidase
[72]	Assess the prevalence of Fabry disease	933 dialysis patients (557 males [59.7%] and 376 females [40.3%])	Filter paper	α‐Gal A activity can be assessed using DBS samples
[73]	Determine the prevalence of Fabry disease within patients diagnosed with ESRD	142 adult patients (81 males [57.0%] and 61 females [43.0%])	Filter paper	α‐Gal A activity can be assessed using DBS samples
[74]	Screen for Fabry disease in proteinuric patients with Fabry nephropathy	53 patients (39.94 ± 11.9) (37 males [69.8%] and 16 females [30.2%])	Filter paper	DBS samples can be used to analyse genomic DNA (GLA gene specifically) and α‐Gal A activity
[75]	Determine whether DBS samples can be used to monitor the presence of hepatitis B virus markers	430 adult patients with HIV, coagulopathies and CKD	Filter paper	Demonstrated that DBS can be used to detect (with high sensitivity) the presence of HBsAg, anti‐HCV and anti‐HBc
[76]	In future, identify the prevalence of subclinical atheromatous disease and hidden kidney disease	19,800 patients with at least one cardiovascular disease risk factor	Filter paper	DBS will be used for creatinine, uric acid, total cholesterol and lipid profiles
[77]	Identify the possibility of using salivary urea to diagnose and stage CKD	20 participants (13 males [65.0%] and 7 females [35.0%])	ATR crystal	Dried saliva samples can be used to determine urea concentrations
[26]	Determine the accuracy of using dried urine spot samples to measure urinary β2MG/Cr ratio	2623 3‐year‐old patients	Filter paper DUS	DUS samples can be used to measure the levels of both creatinine and β2MG in a fast and simple manner
[78]	Estimate the frequency of APOL1 risk alleles in patients with CKD	48 participants (median = 51.5) (16 males [33.3%] and 32 females [66.7%])	Filter paper	Able to extract DNA from the DBS samples to genotype for two APOL1 risk alleles (G1 and G2)

Abbreviations: anti‐HBc, antibody to hepatitis B core antigen; ATR, attenuated auto reflectance; CKD, chronic kidney disease; Cr, creatinine; DBS, dried blood spot; DNA, deoxyribonucleic acid; DUS, dried urine spot; ESRD, end‐stage renal disease; FSGS, focal segmental glomerulosclerosis; GFR, glomerular filtration rate; HBsAg, hepatitis BS surface antigen; HCV, hepatitis C virus.

##### 3.3.3.3 Biomarker Discovery

Three studies (4.5%) focused upon the identification of disease‐specific biomarkers. In two instances, this was determined using DBS to quantify the metabolomic differences associated with either cystic kidney disease or hypertension, where 220 metabolites could be identified from a single DBS [79, 80]. These metabolites encompassed amino acids, organic acids, bile acids, sugars, acylcarnitines, neurotransmitters, polyamines and steroids [79]. Furthermore, glutaryl carnitine was found to be a potential diagnostic biomarker of kidney failure [81].

##### 3.3.3.4 Disease Monitoring and Screening

The feasibility of disease monitoring by analysing dried biofluids (blood [*n* = 21, 31%], urine [*n* = 2, 3%] and saliva [*n* = 1, 1.5%]) was assessed by 23 studies (34.3%). The majority reported on Fabry disease to determine its prevalence within a given population via an enzymatic activity assay of α‐galactosidase A (α‐Gal‐A) (*n* = 16) [59, 60, 61, 62, 63, 64, 65, 66, 67, 68, 69, 70, 71, 72, 73, 74]. Glycosphingolipids, globotriaosylceramide and globotriaosylsphingosine are downstream markers of α‐Gal‐A deficiency and were monitored with microsampling individually [64, 65, 66, 68, 71] or simultaneously with creatinine measurement in urine specimens [68]. Genetic screening for GLA gene mutations was also performed using DBS in four studies (6%) [63, 65, 68, 74].

In three studies, dried biofluids were used to screen or monitor chronic kidney disease. One study screened the genetic risk factor APOL1, and 19.4 ng of DNA could be extracted from each DBS [78]. In addition to genetic screening, the concentrations of salivary urea and creatinine were assessed [58, 77] using the DBS method, and it was able to distinguish healthy controls from patients with CKD Stages 3–5 but not those with CKD Stages 1 and 2 [77]. One further study evaluated the use of DBS to detect the presence of the Hepatitis B and C viruses in patients with CKD by locating HBsAg, anti‐HBc and anti‐HCV in given samples [75].

Using 2813 paediatric patients from two studies, 2623 DUB and 190 DBS samples were used to screen for congenital anomalies of the kidney and the urinary tract [17, 26]. The samples were used to identify creatinine levels and urine β2‐microglobulin (β2‐M), demonstrating that either DUS or DBS were deemed suitable for mass paediatric screening [17, 26]. One utilised tandem mass spectrometry [17] to measure creatinine while the other measured samples either via an enzyme‐linked immunosorbent assay or latex photometric immunoassay for creatinine and β2‐M, respectively [26].

### 3.4 Cost of Using Dried Biofluids

The reduced cost of dried biofluid microsampling is a frequently cited advantage of microsampling. A total of 10 studies (14.9%) stated that dried biofluid microsampling was cheaper relative to traditional venous blood collection [24, 26, 29, 32, 38, 43, 56, 66, 72]. This was due to reduced costs associated with sample preparation, consumables and storage at room temperature [24, 32, 56, 72]. Furthermore, there were significant staff savings [32, 45]. In total, four studies (6%) estimated the actual cost saving associated with the use of DBS. Dickerson et al. [32] reported a saving of $1635 per patient every year for monitoring tacrolimus concentration. One study found that the cost saving is only realised if DBS eliminated a hospital visit, leading to an annual cost saving of €76,608 [43]. Martial et al. [45] performed a cost analysis of DBS compared with conventional sampling, resulting in a 61% societal and 21% healthcare cost saving for patients with kidney disease. Dried urine spots were estimated to cost $6.4/person including consumables, labour and delivery costs [26].

### 3.5 Stability of Dried Biofluids

**FIGURE 2:**
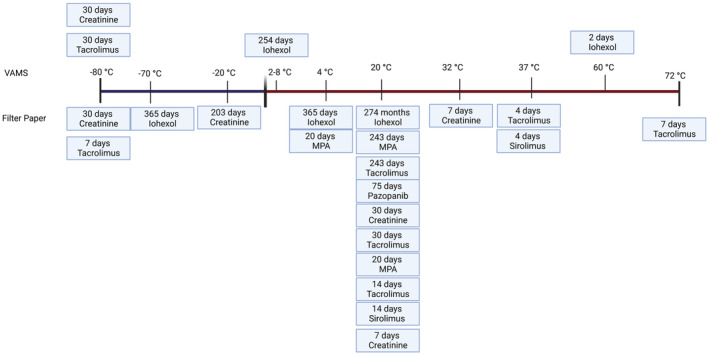
Schematic of the duration of analyte stability when using volumetric absorptive microsampling (VAMS) devices (top row) or dried blood spot (DBS) on filter paper (bottom row) according to storage temperature. Created with BioRender.com.

Surprisingly, only 13 (19%) studies reported on analyte stability according to storage duration and temperature for DBS (Figure 2), and these studies had variable findings and covered six pharmaceutical agents and creatinine. Pazopanib concentrations were stable at room temperature for 75 days [50]. Tacrolimus concentrations were more extensivity studied with room temperature stability being favourable between 2 weeks [32] and 8 months [27, 31], and samples stored at −20°C suitable for up to 29 weeks [19]. One study also found tacrolimus to be stable for 4 days at 37°C [32]. A single‐method development study found that samples stored at −80°C started to degrade within a month [38] whereas another study showed that tacrolimus concentrations were stable for 1 month stored at 72°C [27]. Sirolimus concentrations were stable for 2 weeks [32] at room temperature and 4 days at 37°C [32]. MPA concentrations demonstrated stability at room temperature and 4°C for 20 days [48], and another study found it to be stable at room temperature and 4°C for up to 8 months [27]. Two Iohexol studies showed extremely good term stability with stability for 9 months at room temperature [22], for 254 days to 12 months at 2–8°C [22, 23] and 1 year at −20°C [22]. Iohexol was even stable for 2 days at 60°C [23] and following four freeze‐thaw cycles [22]. Creatinine concentrations were stable at room temperature for 1 month [31], with another study confirming stability at 1 week when stored at room temperature in addition to being stored at 32°C [19]. For samples that were frozen, creatinine was reported to be stable for 29 weeks [19] and for at least 1 month at −80°C [38]. Interestingly, a study by Mathew et al. [38] suggested that stability was dependent upon the type of device being used with Whatman paper and VAMS devices having different thermal stability properties for creatinine and tacrolimus concentrations.

The handling of dried biofluids upon receipt varied between studies, with two studies stating that the samples were stored at room/ambient temperature with a strict time limit of when to perform further analysis, for example 24 or 48 h [28, 49]. Several studies stored dried biofluids long term at room or ambient temperature typically with desiccant to protect the samples from moisture [16, 34, 57, 63, 65, 78]. Some studies applied a time limit for 4°C storage [24] while others suggested the use of 4°C storage long term [27, 42, 72, 73]. The most common option for long‐term storage prior to analysis, as described in 10 papers, was −20°C or −80°C in conjunction with the use of desiccant [15, 18, 38, 47, 50, 51, 58, 64, 69, 79].

### 3.6 Patient Acceptability

The majority of patients favoured microsampling over conventional methods. This was often due to a reduction in pain levels [24] or convenience, for instance reducing the number of hospital visits and/or not having to provide an overnight urine sample [43, 55]. The VAMS device was shown to have significantly higher usability due to patient confidence and device simplicity [29, 36]. One study, using both a visual analogue scale and a nine‐item questionnaire, respectively reported a preference for DBS in 20 caregivers and 25 young people [30]. Another identified that 50% of children would choose a finger prick, 42% preferred a standard blood sample, while 8% had no preference [27].

In the few studies that noted adults favouring traditional samples, the reasons were due to impaired vision and excessive finger squeezing to generate sufficient blood volumes [33]. The paediatric cohort identified potential concerns of fear, pain, sampling, dislike of the needle and dislike of the sound of the automated lancet [27].

## 4 Discussion

The landscape of dried biofluids is rapidly evolving, owing to the fields' growing popularity within a variety of clinical and basic science research areas and at‐home sampling capabilities. Currently, DBS cards are widely used in neonatal screening for a variety of disorders. In recent years, more sensitive bioanalytical methods have emerged allowing more accurate data such as genomic, proteomic and metabolomic approaches, to be obtained from increasingly smaller sample volumes. This is the first systematic review to assess the current uses of biofluid microsampling in patients with kidney disease and outlines important considerations, such as patient acceptability, sample stability and cost, for upscaling this novel technique. In addition, this is the first to review additional dried biofluids, namely DUS and DSS. The non‐invasive biofluid collection offers the opportunity for patients with a fear of needles and those too young to provide finger‐prick blood samples with the opportunity to contribute to research.

In general, dried biofluids demonstrated acceptable specificity and sensitivity when compared to conventional sampling techniques, regardless of the collection method or type of biofluid. This novel methodology offers hope for patients with kidney disorders as it may preserve vein health by avoiding repeated venous sampling [3].

These encouraging findings support the collection of dried biofluids for short‐term and long‐term storage, which leads to the potential of enhanced biobanking abilities for research. In the omics field, long‐term biobanked dried blood and urine samples offer exciting potential for subsequent proteomic analysis of blood [82] and urinary and blood‐based metabolomic [6] analysis.

Patient acceptability indicated increased willingness to participate in microsampling, which could lead to broader patient recruitment and importantly longitudinal sampling [57]. It is worth noting that other studies have highlighted concerns over the quality of instructions with sampling at home [9] with one study providing telephone coaching for sample collection [83]. Recent work has highlighted that the majority of patient information leaflets exceed the average reading level in the UK [84]. Thus, adequate patient involvement during the development of at‐home sampling instructions will be important to ensure high‐quality samples can be collected.

Ultimately, this review identifies areas that are yet to be explored, such as monitoring disease flares in glomerulonephritis, and offers evidence to support the need to expand this area beyond drug monitoring, to improve diagnosis, stratification of patients, disease activity assessment or prognosis. This work demonstrates that further methodological optimisation is required to ensure dried biofluids are truly reflective of venous sampling to maintain the highest degree of accuracy [24]. This was particularly evident with the use of VAMS where they typically performed worse than filter paper dispensed blood spots. It is likely that volumetric devices will become a preferred option for dried biosampling as it eliminates the effect of haematocrit, which is dependent upon the area of the blood spot [85] and provides reproducible sampling [86].

This systematic review has limitations, reflecting the current application of dried biofluids in kidney disease. Blood was by far the most predominant biofluid of interest, meaning that data relating to other biofluids such as urine or saliva samples were severely limited. In addition, we only evaluated dried biofluids from the perspective of health conditions related to kidneys, and DBS have already been applied to a wide range of cancer [87], environmental exposure monitoring [88] and cardiovascular disease [89] to name just a few. This provides additional support to the adoption of dried biofluids for kidney research.

Overall, this review highlights the use of microsampling for kidney disease and compelling evidence to expand beyond its current applications. The first step in advancing this is to develop a standard operating procedure after a process of method optimisation followed by further cohort studies using patients with matched clinical data and analytes of clinical significance. Subsequent upscaling could be conducted in a controlled manner if appropriate with patient engagement to ensure feasibility and success. It is important that advances such as dried biofluids are applied to kidney disease as it is typically a silent overlooked disease despite its significant health and economic costs [90, 91]. The ability to include greater numbers of patients and collect longitudinal studies may help to allay the significant projected costs associated with the increase of kidney disease worldwide.

## 5 Conclusion

Dried biofluids have been used in a variety of areas within the kidney field, with the majority focused on the monitoring of immunosuppressive agents. This work shows that it is important to consider more than just DBS as potential research solution in kidney disease. Further work is required to validate these approaches within the broader field to advance mechanistic insights into kidney disease by linking the state‐of‐the‐art science to the patient's home.

## Consent

No patients were recruited as the study is a secondary review of the existing literature.

## Conflicts of Interest

The authors declare no conflicts of interest.

## Data Availability

Data sharing is not applicable as no new data are generated.
